# Supplementary material for: An Extended Admixture Pulse Model Reveals the Limitations to Human–Neandertal Introgression Dating
Source: Mol Biol Evol. 2021 Jul 12;38(11):5156–74. doi: 10.1093/molbev/msab210 (PMC8557420; doi:10.1093/molbev/msab210)
Supplement: msab210_Supplementary_Data [file msab210_supplementary_data.zip › An_extended_admixture_pulse_model_reveals_the_limitations_to_Human-Neandertal_introgression_dating_supplements.pdf]

An extended admixture pulse model reveals the limitations to Human-Neandertal introgression dating

Iasi, Leonardo N. M., Ringbauer, Harald and Peter , Benjamin M.

Supplement Material

Supplement Figures

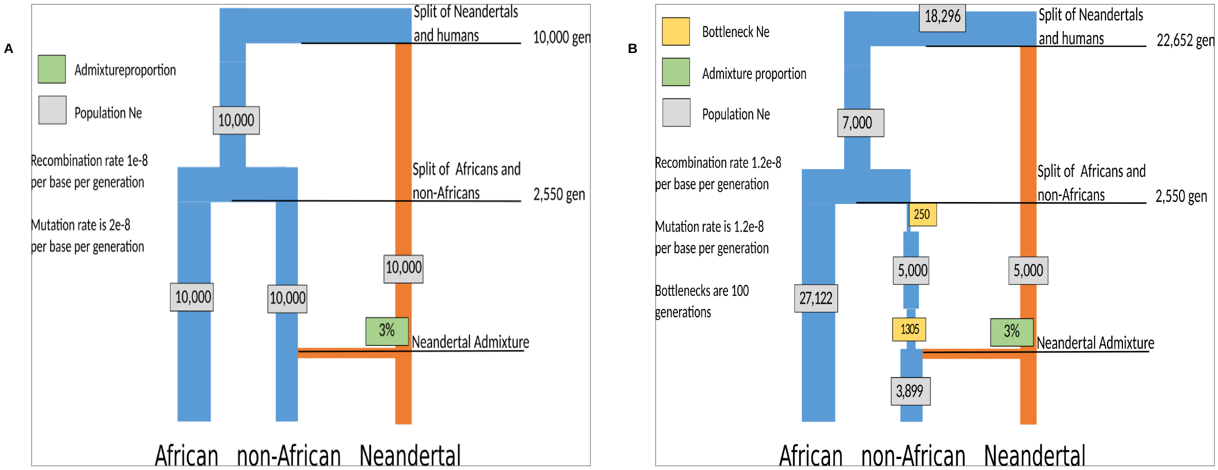

Figure 1: Demographic models of Neandertal introgression into non-Africans used for the simulations. A) Simple demographic model used for ALD simulations with constant population sizes. B) Complex demographic model with population size changes and split times similar to Skov et al. 2018.

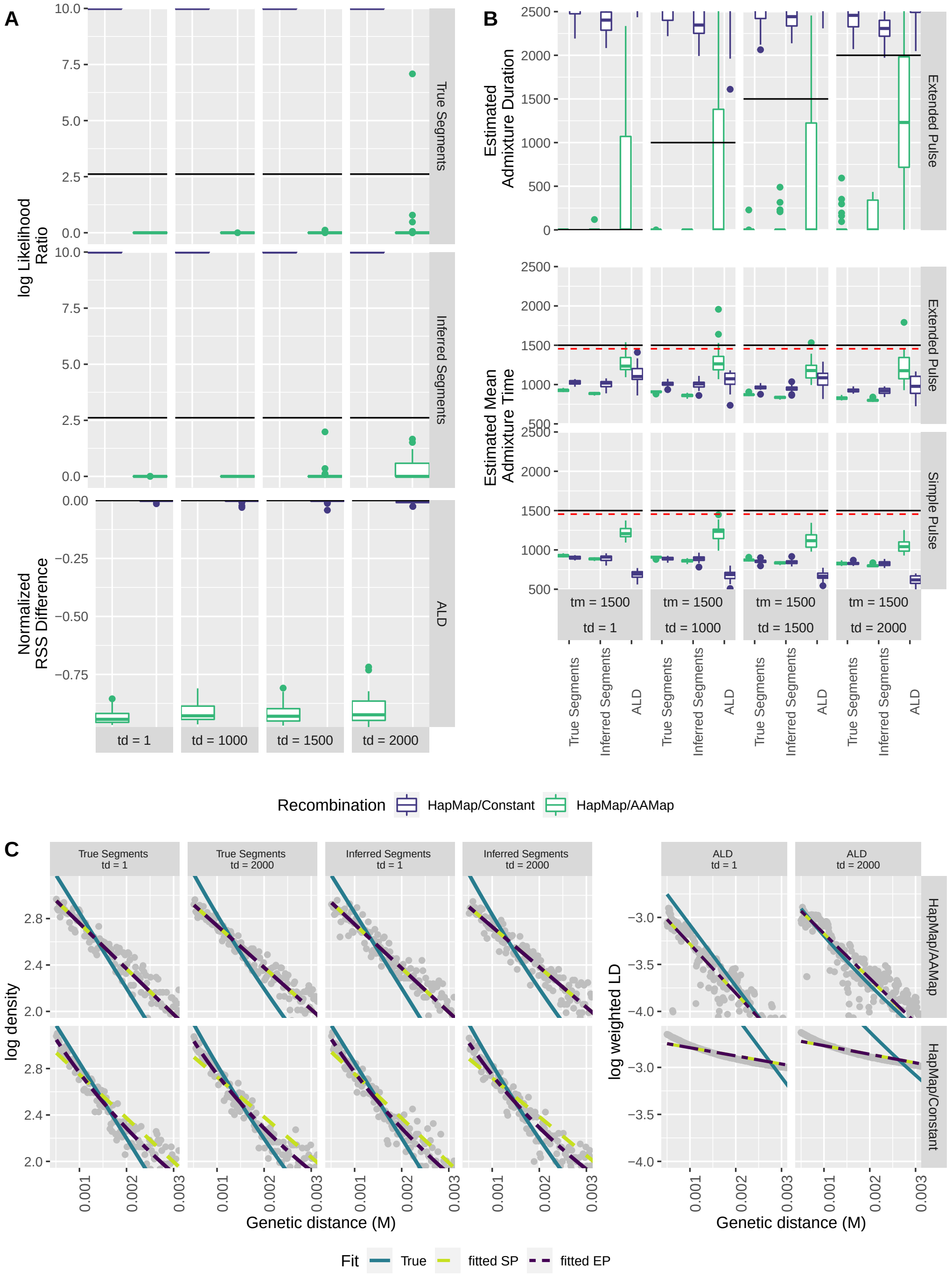

Figure 2: Comparison between the simple and extended pulse on true and inferred segment length and ALD decay estimates using an empirical recombination map (HapMap) for simulations with a fixed mean time (t_m) of 1,500 generations ago and varying durations (t_d). Genetic distances are assigned either using a constant rate (HapMap/Constant) or the AAMap (HapMap/AAMap). All times are given in generations. A) Log likelihood ratios between the two models for segment data and normalized difference between the residual sum-of-squares between the two models for ALD data. B) Mean time estimates of admixture and extended pulse estimate for admixture duration. Solid black line indicates true t_m and t_d, red dotted line indicates migration corrected admixture time (t_m(1-m)). C) Comparison of the fit to data between the simple and extended pulse using true and estimated parameters.

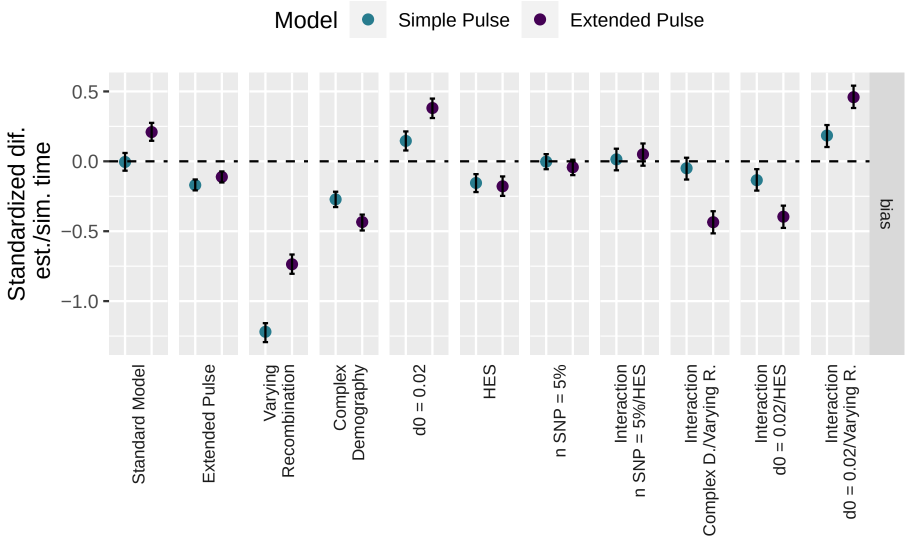

Figure 3: GLM effect sizes for the deviation between simulated and estimated mean admixture time and 95% C.I. for the parameters between the simple and extended pulse model: gene flow (simple/extended), recombination rate (constant/varying), demography (simple/complex), minimal genetic distance (0.02/0.05 cM), SNPs used for ALD calculation (100 % / 5 %) and ascertainment scheme (LES/HES). Estimates are calculated across all possible combinations of parameters. Dotted horizontal line indicates unbiased admixture estimates.

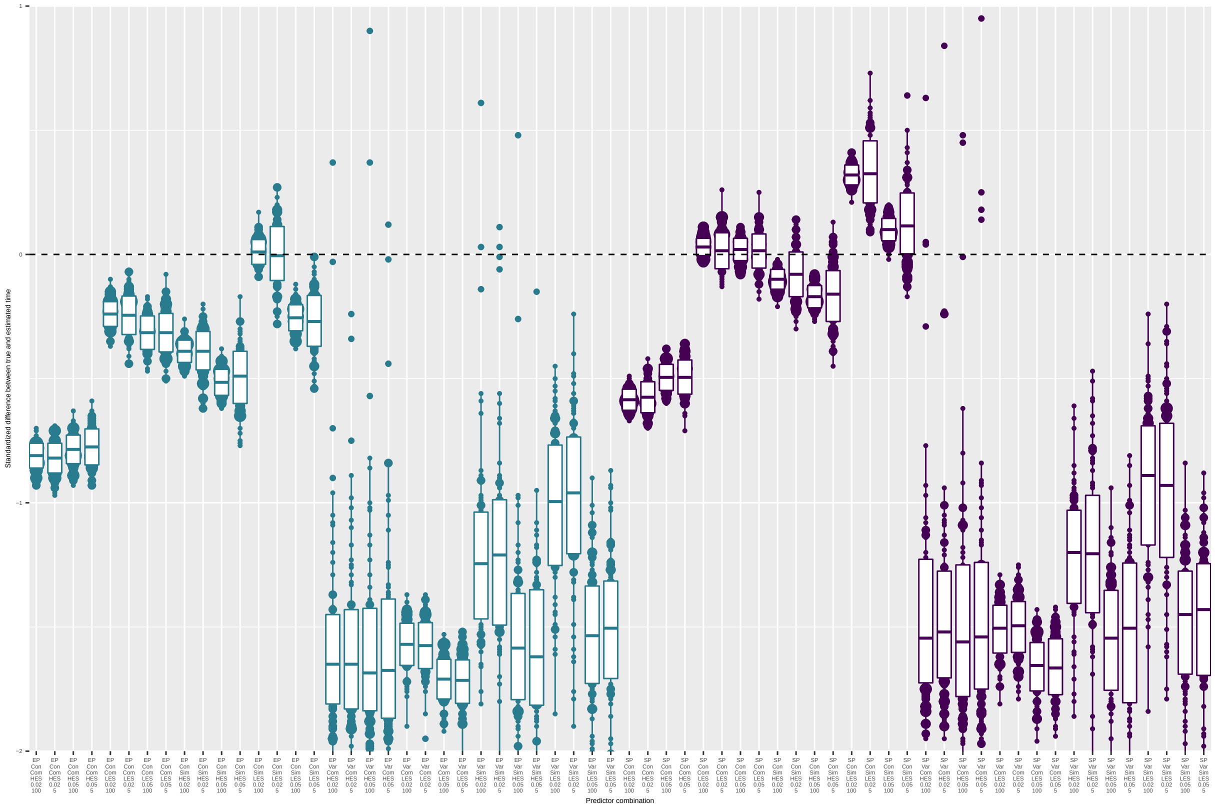

Figure 4: Comparison of the standardized difference between true and estimated mean admixture time under the simple pulse model for simulations of all combinations of parameters: ascertainment scheme = LES/HES, minimal genetic distance (d_0) = 0.02/0.05 cM, demography = simple/complex (sim/com), recombination = constant/variable (con/var), SNP used 100 % / 5 % and the gene flow model = simple pulse/extended pulse (SP/EP). Simulations with a simple pulse gene flow are indicated in purple, extended pulse in turquoise. Each simulation was repeated 100 times. Dotted horizontal line indicates no difference between true and estimated time.

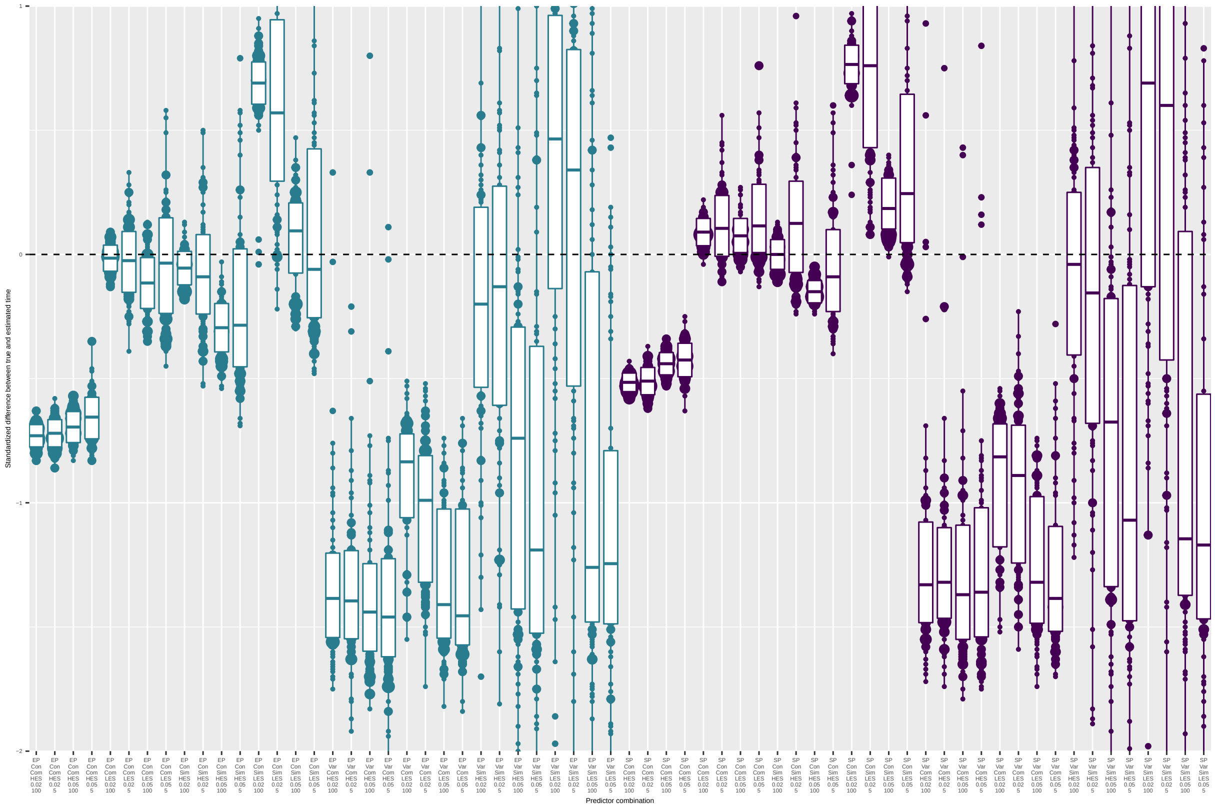

Figure 5: Comparison of the standardized difference between true and estimated mean admixture time under the extended pulse model for simulations of all combinations of parameters: ascertainment scheme = LES/HES, minimal genetic distance (d_0) = 0.02/0.05 cM, demography = simple/complex (sim/com), recombination = constant/variable (con/var), SNP used 100 % / 5 % and the gene flow = simple pulse/extended pulse (SP/EP). Simulations with a simple pulse gene flow are indicated in purple, extended pulse in turquoise. Each simulation was repeated 100 times. Dotted horizontal line indicates no difference between true and estimated time.

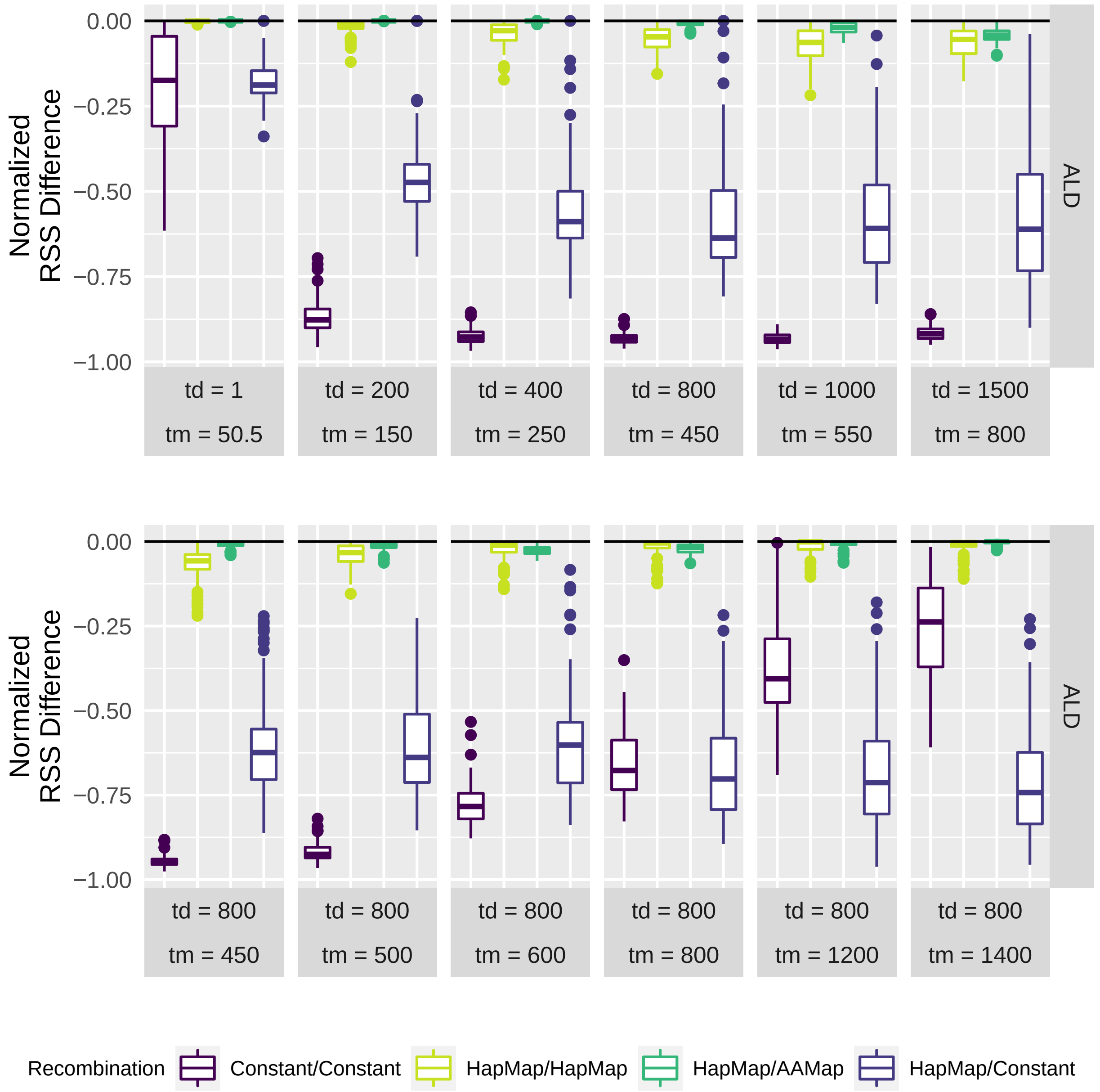

Figure 6: Sampling closer to the admixture event: Normalized difference in the residual-sum-of-squares between the simple pulse fit and extended pulse fit A) Comparison for scenarios sampled 50 generations after the gene flow ended, with different gene flow durations. B) Comparison for scenarios with different sampling times after 800 generations of gene flow.

Supplement Tables

Table 1: Comparing effect sizes for technical covariates: GLM parameter estimates on the bias between the true and estimated mean admixture time

model	GLM	variable	mean	5.5%	94.5%	n eff	Rhat
Simple Pulse	bias	Standard Model	0.00	-0.07	0.06	1922	1
	bias	Extended Pulse	-0.17	-0.21	-0.13	2439	1
	bias	Varying Recombination	-1.22	-1.29	-1.16	2521	1
	bias	Complex Demography	-0.27	-0.33	-0.22	2488	1
	bias	d0 = 0.02	0.15	0.08	0.21	2282	1
	bias	HES	-0.15	-0.22	-0.09	2507	1
	bias	n SNP = 5%	0.00	-0.06	0.05	3171	1
	bias	Interaction n SNP = 5%/HES	0.01	-0.06	0.09	2169	1
	bias	Interaction Complex D./Varying R.	-0.05	-0.13	0.03	2386	1
	bias	Interaction d0 = 0.02/HES	-0.14	-0.21	-0.06	2032	1
	bias	Interaction d0 = 0.02/Varying R.	0.18	0.10	0.26	2086	1
	bias	Sigma	0.79	0.77	0.80	4267	1
Extended Pulse tm	bias	Standard Model	0.21	0.15	0.28	1876	1
	bias	Extended Pulse	-0.11	-0.15	-0.07	2650	1
	bias	Varying Recombination	-0.74	-0.80	-0.67	2284	1
	bias	Complex Demography	-0.43	-0.49	-0.38	2678	1
	bias	d0 = 0.02	0.38	0.31	0.45	2330	1
	bias	HES	-0.18	-0.25	-0.11	2556	1
	bias	n SNP = 5%	-0.04	-0.10	0.01	3891	1
	bias	Interaction n SNP = 5%/HES	0.05	-0.03	0.13	2196	1
	bias	Interaction Complex D./Varying R.	-0.44	-0.52	-0.36	2375	1
	bias	Interaction d0 = 0.02/HES	-0.40	-0.48	-0.32	2312	1
	bias	Interaction d0 = 0.02/Varying R.	0.46	0.38	0.54	2110	1
	bias	Sigma	0.81	0.79	0.82	4128	1

Table 2: Comparing effect sizes for technical covariates: GLM parameter estimates on absolute deviation between the true and estimated mean admixture time

model	GLM	variable	mean	5.5%	94.5%	n eff	Rhat
Simple Pulse	abs. deviation	Standard Model	0.08	0.02	0.14	1880	1
	abs. deviation	Extended Pulse	0.15	0.12	0.19	2456	1
	abs. deviation	Varying Recombination	1.26	1.19	1.32	3091	1
	abs. deviation	Complex Demography	0.17	0.12	0.22	2621	1
	abs. deviation	d0 = 0.02	-0.06	-0.12	0.00	2293	1
	abs. deviation	HES	0.18	0.12	0.24	2347	1
	abs. deviation	n SNP = 5%	0.01	-0.04	0.06	3529	1
	abs. deviation	Interaction n SNP = 5%/HES	0.00	-0.07	0.07	2086	1
	abs. deviation	Interaction Complex D./Varying R.	0.03	-0.03	0.11	2728	1
	abs. deviation	Interaction d0 = 0.02/HES	0.10	0.03	0.17	2336	1
	abs. deviation	Interaction d0 = 0.02/Varying R.	-0.23	-0.30	-0.16	2206	1
	abs. deviation	Sigma	0.71	0.70	0.72	4341	1
Extended Pulse tm	abs. deviation	Standard Model	0.15	0.09	0.20	2138	1
	abs. deviation	Extended Pulse	0.09	0.05	0.12	3011	1
	abs. deviation	Varying Recombination	0.82	0.76	0.88	2697	1
	abs. deviation	Complex Demography	0.02	-0.03	0.07	2665	1
	abs. deviation	d0 = 0.02	0.11	0.05	0.17	2746	1
	abs. deviation	HES	0.10	0.04	0.16	2804	1
	abs. deviation	n SNP = 5%	0.07	0.02	0.12	4095	1
	abs. deviation	Interaction n SNP = 5%/HES	0.01	-0.06	0.08	2698	1
	abs. deviation	Interaction Complex D./Varying R.	0.28	0.21	0.35	2705	1
	abs. deviation	Interaction d0 = 0.02/HES	-0.03	-0.10	0.04	2535	1
	abs. deviation	Interaction d0 = 0.02/Varying R.	-0.41	-0.48	-0.34	2392	1
	abs. deviation	Sigma	0.71	0.70	0.72	4063	1

Table 3: Application to Neandertal data: Estimates

	Model	tm	A	c
Simple Pulse	Simple Pulse	1682 (1526 - 1839)	0.019 (0.019 - 0.019)	0.000182 (0.00016 - 0.000204)
Extended Pulse (td=1)	Extended Pulse (td=1)	1682 (1526 - 1839)	0.019 (0.019 - 0.019)	0.000182 (0.00016 - 0.000204)
Extended Pulse (td=100)	Extended Pulse (td=100)	1683 (1527 - 1839)	0.019 (0.019 - 0.019)	0.000182 (0.00016 - 0.000204)
Extended Pulse (td=200)	Extended Pulse (td=200)	1685 (1528 - 1841)	0.019 (0.019 - 0.019)	0.000182 (0.00016 - 0.000204)
Extended Pulse (td=400)	Extended Pulse (td=400)	1691 (1534 - 1848)	0.019 (0.019 - 0.019)	0.00018 (0.000158 - 0.000202)
Extended Pulse (td=800)	Extended Pulse (td=800)	1717 (1558 - 1877)	0.019 (0.019 - 0.019)	0.000174 (0.000152 - 0.000196)
Extended Pulse (td=1000)	Extended Pulse (td=1000)	1736 (1575 - 1898)	0.02 (0.02 - 0.02)	0.00017 (0.000148 - 0.000192)
Extended Pulse (td=1500)	Extended Pulse (td=1500)	1800 (1633 - 1967)	0.02 (0.02 - 0.02)	0.000155 (0.000134 - 0.000177)
Extended Pulse (td=2000)	Extended Pulse (td=2000)	1882 (1707 - 2057)	0.021 (0.021 - 0.021)	0.000138 (0.000116 - 0.000159)
Extended Pulse (td=2500)	Extended Pulse (td=2500)	1978 (1794 - 2161)	0.022 (0.022 - 0.022)	0.000118 (9.6e-05 - 0.00014)

Table 4: Application to Neandertal data: RSS

Model	RSS
Simple Pulse	2.53e-05
Extended Pulse (td=1)	2.53e-05
Extended Pulse (td=100)	2.53e-05
Extended Pulse (td=200)	2.53e-05
Extended Pulse (td=400)	2.53e-05
Extended Pulse (td=800)	2.51e-05
Extended Pulse (td=1000)	2.50e-05
Extended Pulse (td=1500)	2.47e-05
Extended Pulse (td=2000)	2.44e-05
Extended Pulse (td=2500)	2.42e-05
